# Efficient Oral Priming of *Tenebrio molitor* Larvae Using Heat-Inactivated Microorganisms

**DOI:** 10.3390/vaccines10081296

**Published:** 2022-08-11

**Authors:** Sergio González-Acosta, Victoria Baca-González, Patricia Asensio-Calavia, Andrea Otazo-Pérez, Manuel R. López, Antonio Morales-delaNuez, José Manuel Pérez de la Lastra

**Affiliations:** 1 Biotechnology of Macromolecules Research Group, Instituto de Productos Naturales y Agrobiología, (IPNA-CSIC), 38206 San Cristóbal de la Laguna, Spain; 2 Escuela de Doctorado y Estudios de Posgrado, Universidad de La Laguna Avda, Astrofísico Francisco Sánchez, SN. Edificio Calabaza-Apdo, 456, 38200 San Cristóbal de La Laguna, Spain

**Keywords:** immune priming, antimicrobial peptides, invertebrate immunity, mealworms, in vitro antimicrobial assay, survival rate

## Abstract

Microbial resistance is a global health problem that will increase over time. Advances in insect antimicrobial peptides (AMPs) offer a powerful new approach to combat antimicrobial resistance. Invertebrates represent a rich group of animals for the discovery of new antimicrobial agents due to their high diversity and the presence of adaptive immunity or “immune priming”. Here, we report a priming approach for *Tenebrio molitor* that simulates natural infection via the oral route. This oral administration has the advantage of minimizing the stress caused by conventional priming techniques and could be a viable method for mealworm immunity studies. When using inactivated microorganisms for oral priming, our results showed an increased survival of *T. molitor* larvae after exposure to various pathogens. This finding was consistent with the induction of antimicrobial activity in the hemolymph of primed larvae. Interestingly, the hemolymph of larvae orally primed with *Escherichia coli* showed constitutive activity against *Staphylococcus aureus* and heterologous activity for other Gram-negative bacteria, such as *Salmonella enterica*. The priming of *T. molitor* is generally performed via injection of the microorganism. To our knowledge, this is the first report describing the oral administration of heat-inactivated microorganisms for priming mealworms. This technique has the advantage of reducing the stress that occurs with the conventional methods for priming vertebrates.

## 1. Introduction

Invertebrates form the largest and most diverse group of the animal kingdom, making up 95% of the fauna. They have survived for millions of years. Much of their success is due to a rapid and highly efficient immune response that detects, inactivates, and ultimately removes pathogens from their environment. Some insects, such as cockroaches, live in polluted environments contaminated with multidrug-resistant pathogens. Insects can provide an almost unlimited supply of physiologically active chemicals. Insect-derived natural products have long been used in traditional medicine, are considered a source of new antimicrobial molecules with agricultural and therapeutic benefits [1,2], and continue to be an important source of therapeutic ingredients in developing countries. The immune system of insects is divided into a cellular and a humoral response, and they share several conserved features of the innate immune system with vertebrates [3]. The innate immunity of vertebrates is extremely sensitive to bacterial challenges. As part of its defense mechanism, it produces humoral antimicrobial substances in response to bacterial attacks. The venoms of some invertebrates such as scorpions, spiders, and ants also have useful antibacterial properties [4]. This led to the idea that invertebrates have a strong antimicrobial defense system that can be explored for the discovery of new antibacterial agents.

Antimicrobial peptides (AMPs) are multifunctional components of the immune defense system used by prokaryotic and eukaryotic organisms [5]. AMPs have remained active throughout evolution, have produced little or no resistance, and could therefore be an alternative to conventional antibiotics. There are a growing number of studies identifying invertebrate AMPs that can be used against bacteria, fungi, and even viruses [6]. They may also have anticancer properties, with high selectivity and efficacy on cancer cells, which has led to their designation as anticancer peptides (ACPs) [7]. As a result, AMPs and ACPs have been the subject of several investigations for the development of new antibiotics against multidrug-resistant bacteria (MDR), as well as new anticancer drugs [8]. AMPs are typically cationic and amphipathic and have a structure that contains both hydrophobic and hydrophilic moieties with a net charge of +2 to +9 and a low molecular weight (12–50 amino acids) [9]. They are effective against a variety of pathogens, including antibiotic-resistant bacteria.

The humoral response of insects includes activation of the proPO system (PO), increase in reactive oxygen species, and synthesis of AMPs [10]. In insects, AMPs are synthesized by hemocytes and other cells such as epidermal epithelial cells or fat body cells, which release these peptides into the hemolymph [11]. Toll and Imd are the major signaling pathways for the production of AMP in insects [12], while the Jak-Stat and JNK signaling pathways may also be involved through other complementary functions [13]. The activation of each signaling pathway depends on the type of microorganism eliciting the response [14], but they may also interact and act synergistically [15]. Gram-positive bacteria or fungi have been reported to trigger activation of the Toll pathway [16,17]. Gram-negative bacteria, on the other hand, activate the Imd pathway [18] and, alternatively, the JNK pathway, all of which leads to the production of AMPs [19].

Due to the absence of lymphocytes, it has been suggested that invertebrates lack an adaptive immune system [20,21]. However, certain insects exhibit a phenomenon known as immunological priming, in which previous exposure to a sublethal dose of a pathogen or pathogen-derived material leads to an increase in the immune response, making the insect resistant to a future lethal infection [13,22,23,24,25]. This concept has been defined as the ability of the insect immune system to retain and reuse information about molecular patterns to enhance the immune response to successive challenges [14,26]. The specificity of the basic response represents the ability to discriminate between different challenges [27].

In the absence of a conventional model, there is a wide variability in the available literature in the methodology describing priming to induce AMPs, which differs mainly in four factors: invertebrate species, developmental stage, immunogen used, and priming method [28]. Furthermore, the induction of AMPs can be transmitted to offspring in a process called transgenerational immune priming [29,30]. The species tested can be selected for ease of breeding and handling, such as *Tenebrio molitor* or *Galleria mellonella* [31,32,33], or for other characteristics, such as a fully sequenced genome, as in the case of *Tenebrio molitor* or *Bombix morii* [34,35]. The developmental stage is important because of differences in the physiology, metabolism, and immunity between stages. The choice of the specific developmental stage for priming can be influenced by the surrounding microorganisms and the rearing and handling capabilities of the invertebrates [28]. The immunogen used also determines the specificity and duration of the response [24]. Many studies have used a low concentration of live microorganisms as the immunogen, inoculating a nonlethal dose to elicit the appropriate baseline response [33,36,37]. However, other studies have described the inoculation of live microorganisms at higher concentrations that reproduce natural infection [38,39] without invertebrate mortality [40]. Priming with inactivated microorganisms can inoculate a high concentration of the immunogen, avoiding the risk of mortality. This method could be advantageous, because immunization is dose-dependent [41]. However, the technique used for inactivation (usually formalin or heat) may interfere with the priming [42]. Microorganism-derived compounds such as peptidoglycan or lipopolysaccharide are also used as immunogens [43,44]. The most common priming method for inducing AMPs in invertebrates is injection and puncture [45,46,47], with some authors reporting the use of oral administration of immunogens [48,49,50] while other authors report the administration of immunogens, especially in terrestrial insects [51]. Each method has advantages and disadvantages. For example, injection and pricking ensure that the immunogen is delivered to the insect body at the administered dose [52]. However, these methods can cause stress due to the injury and handling of the insects, which can lead to artifacts in the results. Several studies have found that stress can cause a priming effect in invertebrates [53,54]. Priming by the oral administration of immunogens is easy to perform and avoids the stress to invertebrates that the other methods can cause. However, this method can be more expensive, and the exact dose for priming can be uncertain, although it can simulate a natural infection [48,55].

The aim of this study was to test the priming of mealworms by the oral administration of heat-inactivated microorganisms to induce a resistance to bacteria and fungi. To our knowledge, there are no previous references in the scientific literature for oral priming in *T. molitor*.

## 2. Materials and Methods

### 2.1. Insect Cultures

Larvae of *Tenebrio molitor* were obtained from a commercial supplier and maintained in well-ventilated plastic boxes at 22 °C in darkness. Prior to experiments, insects were fed bread ad libitum and supplemented with fresh carrot twice per week. Healthy larvae at the 13th–15th instars larger than 2 cm were used for the experiments.

### 2.2. Culture of Microorganisms

Microorganisms were purchased from the Spanish Type Culture Collection (CECT): *Escherichia coli* (CECT 434), *Staphylococcus aureus* (CECT 794), *Candida albicans* (CECT 1392), *Salmonella enterica* (CECT 456), and *Botrytis cinerea* (CECT 20973). The bacteria were cultured in Mueller–Hinton medium and the yeast in Saboraud medium overnight at 37 °C. Meanwhile, *B. cinerea* was grown on tomato agar (25% liquefied tomato fruit, 1.5% agar, pH 5.5) in Petri dishes at 23 °C. On the third day, they were irradiated with ultraviolet light overnight to promote the sporulation of conidia. After 10 days of incubation, the conidia were extracted with 5 mL of distilled water, and the culture surface was scraped. The conidial suspension was then filtered to remove the mycelium, and the concentration was determined microscopically using a hemocytometer [56]. All microorganisms were diluted at 1 × 10^6^ CFU/mL in PBS. For heat inactivation, the microorganisms were autoclaved at 121 °C for 15 min. The success of the inactivation of the microorganisms was tested by plating in the appropriate media and incubation for 24 h at 37 °C (*E. coli*, *S. aureus*, and *C. albicans*) and 48 h at 23 °C (*B. cinerea*).

### 2.3. Priming and Challenge

Oral priming was performed by mixing 2 g of carrots with 1 mL of heat-inactivated microorganisms. Larvae were fed only this mixture ad libitum for 24 h. The challenge procedure consisted of an injection method: first, the larvae were anesthetized by cooling them on ice for 10 min. Then, using a 0.3-mL insulin syringe with a 30G needle, they were injected with 5 µL of a suspension containing live microorganisms (5 × 10^3^ CFU) between the 3rd and 5th instars.

### 2.4. Survival Assay

The larvae were orally inoculated for 24 h and then infected with microorganisms, as previously described. The larvae (*N* = 619) were divided into 9 groups. The treatment of each group is shown in Table 1. 

The group treated with PBS served as a control group to study the effects of the injection procedure on larval survival. The four groups labeled PBS-EC, PBS-SA, PBS-CA, and PBS-BC corresponded to mealworms fed with a mixture of PBS–carrot and treated with 5 µL of the corresponding live microorganisms, as described above. These four groups allowed us to study only the effects of the challenge on larval survival. Finally, to evaluate the effect of oral priming on larval survival, the groups EC-EC, SA-SA, CA-CA, and BC-BC were orally primed with a mixture of inactivated microorganisms and carrots and then challenged with the appropriate live microorganisms used for priming. Larval survival was assessed hourly for 48 h after provocation. Larvae were considered alive if they moved in response to contact.

### 2.5. Hemolymph Collection and Antimicrobial Activity

Hemolymph from the surviving larvae was extracted using the method described by Tabunoki et al. [57], with modifications. Briefly, a hole was pierced in the bottom of a 0.5-mL tube with a 32G needle, and the cap was removed. This 0.5-mL tube was placed in a 1.5-mL tube containing 5 µL of Alsever’s solution (2.05% dextrose, 0.8% sodium citrate, 0.055% citric acid, and 0.42% sodium chloride) to prevent melanization of the hemolymph. These tubes were stored on ice until use. The larvae were chilled on ice for 10 min, and then, one leg was torn off the body. Immediately, the larvae were placed in the upper ice-cooled tube and centrifuged at 100× *g* for 5 min. After centrifugation, Alsever’s solution was added to the collected hemolymph from the lower collection tube until a volume:volume ratio of 1:1 was achieved. Finally, the samples were centrifuged at 13,300× *g* for 10 min to remove cell debris. The supernatant was transferred to a clean 1.5-mL collection tube and stored at −20 °C until the antimicrobial analyses were performed.

After thawing at 4 °C, the hemolymph samples were tested against 5 microorganisms (*E. coli*, *S. aureus*, *S. enterica*, *C. albicans*, and *B. cinerea*). Briefly, bacteria were spread on Mueller–Hinton agar (Saboraud agar was used for fungi) and incubated at 37 °C (28 °C for fungi) for 30 min. Then, 1 µL of each hemolymph sample was added to the Petri dish to test for antimicrobial activity. In addition, 1 µL of the Alsever’s solution was used as a negative control. As a positive control, 1 µL of the specific antibiotic/antimycotic (tetracycline 30 mg/mL for *E. coli* and *S. enterica*, ampicillin 20 mg/mL for *S. aureus*, amphotericin B 2.5 µg/mL for *C. albicans*, and itraconazole 4 mg/mL for *B. cinerea*) was used.

### 2.6. Statistical Analyses

The SPSS Statistics 26 software package (SPSS Inc., Chicago, IL, USA) was used for the statistical analysis of larval survival. Time-dependent larval survival was estimated using the Kaplan–Meier method, and comparisons between curves were determined using Cox regression [58]. The significance level was set at *p* < 0.05.

## 3. Results

### 3.1. Survival Assay

**Figure 1:**
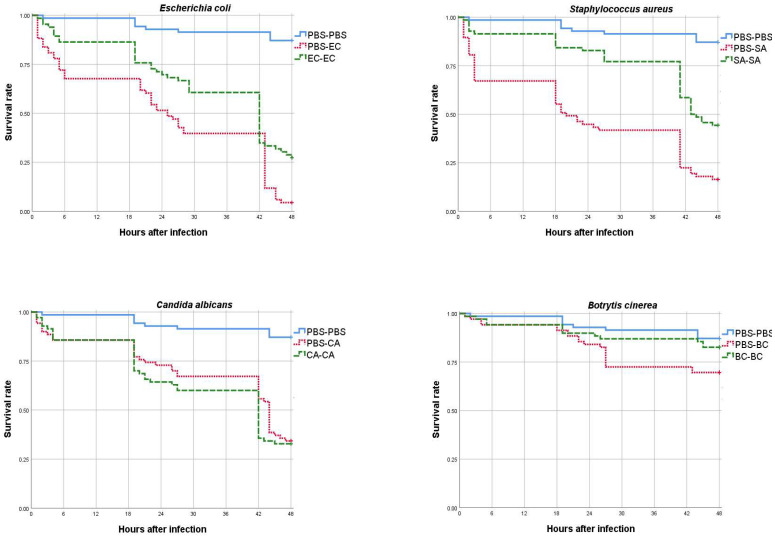
Survival rates (number of alive/total individuals) of *T. molitor* larvae challenged with four microorganisms after priming procedures. (PBS-PBS) Control group, (PBS-EC) larvae primed with PBS and challenged with *E. coli*, (EC-EC) larvae primed and challenged with *E. coli*, (PBS-SA) larvae primed with PBS and challenged with *S. aureus*, (SA-SA) larvae primed and challenged with *S. aureus*, (PBS-CA) larvae primed with PBS and challenged with *C. albicans*, (CA-CA) larvae primed and challenged with *C. albicans*, (PBS-BC) larvae primed with PBS and challenged with *B. cinerea*, and (BC-BC) larvae primed and challenged with *B. cinerea*.

According to the results of the 48-h observation, the larvae of groups EC-EC and SA-SA, which were primed with heat-inactivated Escherichia coli and Staphylococcus aureus and then infected with the respective bacteria, had a higher survival rate than the nonprimed larvae of groups PBS-EC and PBS-SA. The Cox regression and *p*-values of the statistical analysis were 0.004 and <0.001, respectively (Table 2 and Figure 1). The median survival time of the nonprimed groups ranged from 20 to 25 h after the challenge, whereas the median survival time of the orally primed larvae ranged from 42 to 43 h. Thus, oral priming with *E. coli* and *S. aureus* proved effective in protecting mealworms from these microorganisms (Table 2 and Figure 1). For larvae primed and challenged with *C. albicans* (CA-CA) and *B. cinerea* (BC-BC), the survival assay showed that priming did not improve survival (*p* = 0.518 and *p* = 0.086, respectively). As for the effect of challenge compared with the control group (PBS-PBS), Cox regression showed that all microorganisms tested increased the mortality rate of *T. molitor* larvae regardless of prior priming, with the exception of larvae primed with *B. cinerea*. We could not calculate the median for the latter microorganism, because the mortality in this group did not exceed 50% of the initial population.

### 3.2. Antimicrobial Activity

In an attempt to correlate the survival of orally primed larvae of *T. molitor* to a possible higher expression of antimicrobial peptides, we extracted their hemolymphs and used them for in vitro assays against a range of microorganisms, including those used for the priming and challenge. According to us, all hemolymph samples, including those from the control group (PBS-PBS), showed antimicrobial activity against *S. aureus*. However, only the samples from larvae primed and challenged with *E. coli* (group designated EC-EC) showed inhibitory activity against this microorganism in vitro (Table 3). According to the in vitro antimicrobial assay, the hemolymph samples of larvae from the groups EC-EC, SA-SA, and PBS-SA also showed partial antimicrobial activity against *S. enterica*, whereas the hemolymphs of larvae from the groups CA-CA and BC-BC did not exhibit antimicrobial activity against the respective fungal microorganisms: *C. albicans* and *B. cinerea*.

## 4. Discussion

For many years, the absence of lymphocytes in invertebrates led to the conclusion that only an innate immune system was present in this group of animals [21,59]. However, in recent decades, there have been reports of the presence of an inducible immune response that may be specific [13,27]. This immune response is linked to the previous interaction with the pathogen and allows the invertebrate to retain and use this information in a subsequent encounter with the pathogen. However, the specificity of this inducible response, as well as the extent to which it is induced, differs significantly between different invertebrates [60]. Mealworms have been proposed as an optimal model for the pathogenesis of fungal infections, in contrast to other common insects such as *G. mellonella*, *D. melanogaster*, or *B. morii*. Mealworms are easy to keep, which makes them suitable for breeding and the fact that their transcriptome is known [31].

Septic injury is the most commonly used method for priming [28]. Our results support the notion that the oral administration of microorganisms may be an optimal priming method for *T. molitor*. This oral administration mimics a natural pattern. It also avoids the physical stress resulting from injury. The stress induced by priming may alter or modify the observed immune response in these animals [53]. Oral priming has been reported in some insect models, such as *Tribolium castaneum*, *B. morii*, *G. mellonella*, and *Parasemia plantaginis* [48,49,50,61]. However, this method of priming has been little used in research, likely due to the potential interaction of this method with the gut microbiota of insects [62], while it has been reported that the diversity of the mealworm microbiota is low, as it predominantly harbors species of the genus *Spiroplasma* [63].

The heterologous combinations of microorganisms used for priming and challenge can lead to the induction of AMPs [64]. For example, mealworms were found to have increased resistance to fungal infections after the administration of LPS prior to a challenge [58]. Our results showed that the oral administration of *E. coli* and *S. aureus* significantly increased the survival of larvae challenged with a homologous combination of microorganisms. However, we did not observe the same effect when the larvae were primed and challenged with fungi. AMPs could be responsible for the observed antimicrobial activity. The immune response could be related to AMPs, phagocytosis, or PO [10,65]. In our study, the larval group with higher survival rates was consistent with the observed antimicrobial activity of their hemolymph in vitro. Recent results showed that priming insects with bacteria resulted in an increased expression of AMPs such as tenecin, attacin, cecropin, or coleoptericin [10]. Our results suggest that oral priming with inactivated *E. coli* can induce AMPs synthesis via the Imd pathway, leading to activity against Gram-negative bacteria. This may be related to attacin, an inducible AMP expressed against this group of bacteria [66].

The observed antimicrobial response of *T. molitor* hemolymph against *S. aureus* may be due to the production of tenecin-1, an AMP with specific activity against Gram-positive microorganisms. It is described that the stress induced by the challenge injection often leads to the induction of tenecin-1 [67]. The infection of mealworms with *Beauveria bassiana* activated the Toll signaling pathway and induced the expression of some antimicrobial peptides, such as defensin tenecin-1 and coleoptericin tenecin-2 [10]. Moreover, constitutively expressed tenecin-3 showed antifungal activity against *B. bassiana* and protected *T. molitor* from the internal progression of infection by this fungus [68].

Interestingly, the priming and challenge with *E. coli* and *S. aureus* were able to elicit antimicrobial activity against *Salmonella*. This suggests that the antimicrobial activity of their hemolymph could not be specifically induced against this particular pathogen. Moreover, all hemolymph samples, including the control group primed with PBS, showed antimicrobial activity against *S. aureus*, suggesting that the antimicrobial activity might be constitutive against this microorganism. However, we did not grow the mealworms under sterile conditions, and it is possible that *S. aureus* was present in the environment during rearing and before the priming experiments. Nevertheless, it is also possible that the association between our priming experiment and the increase in antimicrobials in the hemolymph of *T. molitor* larvae requires additional inducing factors [53]. In some cases, the increased expression of AMPs is not always associated with a survival advantage for the host [69].

The oral priming of *B. cinerea* showed no significant effect on mealworm survival. With the exception of *S. aureus*, we did not observe any effects of the priming or challenge on antimicrobial activity. In the case of *C. albicans*, there are reports in which tenecin-3 from *T. molitor* showed antifungal activity against this yeast [70]. In the present study, exposure to *C. albicans* increased mealworm mortality. It is possible that the effect of priming was not observed due to the low concentration, as the immune priming response is often dose-dependent [41]. 

## 5. Conclusions

In conclusion, the oral administration of inactivated microorganisms can be considered as an optimal technique for priming *T. molitor*, because it mimics the natural infection of mealworms and avoids the stress that other priming methods based on mechanical manipulations may cause. Oral priming with *E. coli* and *S. aureus* increased the larval survival. In addition, the hemolymph of the surviving larvae showed antimicrobial activity against these microorganisms. Although the challenge with *C. albicans* and *B. cinerea* increased the mortality rate, the hemolymph of the surviving larvae did not show antifungal activity in vitro. 

## Figures and Tables

**Table 1:** Groups of experimental larvae according to the substance used for the priming and challenge. N corresponds with the number of larvae in each group.

Group	Name	Priming	Challenge	*N*
1	Control	PBS	PBS	70
2	PBS-EC	PBS	*E. coli*	68
3	EC-EC	*E. coli*	*E. coli*	66
4	PBS-SA	PBS	*S. aureus*	67
5	SA-SA	*S. aureus*	*S. aureus*	70
6	PBS-CA	PBS	*C. albicans*	70
7	CA-CA	*C. albicans*	*C. albicans*	70
8	PBS-BC	PBS	*B. cinerea*	69
9	BC-BC	*B. cinerea*	*B. cinerea*	69

**Table 2:** Statistical measurements (mean and median) for the groups of *T. molitor* larvae challenged with four microorganisms after the priming procedures. The effects of priming and challenge among the groups were measured by Cox regression and the *p*-values, respectively.

Name	Mean (Hours)	Median (Hours)	Priming	Challenge
PBS-PBS	45.2 ± 1.04	-	-	-
PBS-EC	25.0 ± 2.12	25.0 ± 2.06	0.004	<0.001
EC-EC	33.8 ± 1.94	42.0 ± 2.96		
PBS-SA	24.2 ± 2.25	20.0 ± 2.92	<0.001	<0.001
SA-SA	38.5 ± 1.69	43.0 ± 2.51		
PBS-CA	35.1 ± 1.97	44.0 ± 0.68	0.518	<0.001
CA-CA	32.9 ± 1.96	42.0 ± 3.54		
PBS-BC	40.0 ± 1.64	-	0.086	0.016
BC-BC	43.4 ± 1.45	-		

(PBS-PBS) Control group, (PBS-EC) larvae primed with PBS and challenge with *E. coli*, (EC-EC) larvae primed and challenge with *E. coli*, (PBS-SA) larvae primed with PBS and challenge with *S. aureus*, (SA-SA) larvae primed and challenge with *S. aureus*, (PBS-CA) larvae primed with PBS and challenge with *C. albicans*, (CA-CA) larvae primed and challenge with *C. albicans*, (PBS-BC) larvae primed with PBS and challenge with *B. cinerea*, and (BC-BC) larvae primed and challenge with *B. cinerea*.

**Table 3:** In vitro antimicrobial activity of *T. molitor* hemolymphs against the different microorganisms.

Sample	*Escherichia coli*	*Staphylococcus aureus*	*Candida albicans*	*Botrytis cinerea*	*Salmonella enterica*
PBS-PBS	−/−/−	+/+/+	−/−/−	−/−/−	−/−/−
PBS-EC	−/−/−	P/P/+	−/−/−	−/−/−	−/−/−
EC-EC	P/+/P	+/+/+	−/−/−	−/−/−	P/P/P
PBS-SA	−/−/−	P/P/P	−/−/−	−/−/−	P/P/P
SA-SA	−/−/−	P/P/P	−/−/−	−/−/−	P/P/P
PBS-CA	−/−/−	P/P/P	−/−/−	−/−/−	−/−/−
CA-CA	−/−/−	P/+/P	−/−/−	−/−/−	−/−/−
PBS-BC	−/−/−	+/+/+	−/−/−	−/−/−	−/−/−
BC-BC	−/−/−	P/+/+	−/−/−	−/−/−	−/−/−

(−) Negative, (+) positive, and (P) partial inhibition. (PBS-PBS) Control group, (PBS-EC) larvae primed with PBS and challenge with *E. coli*, (EC-EC) larvae primed and challenge with *E. coli*, (PBS-SA) larvae primed with PBS and challenge with *S. aureus*, (SA-SA) larvae primed and challenge with *S. aureus*, (PBS-CA) larvae primed with PBS and challenge with *C. albicans*, (CA-CA) larvae primed and challenge with *C. albicans*, (PBS-BC) larvae primed with PBS and challenge with *B. cinerea*, and (BC-BC) larvae primed and challenge with *B. cinerea*.

## Data Availability

Not applicable.
